# Physicians’ Mutual Benefit Associaton of Texas

**Published:** 1889-01

**Authors:** 


### PHYSICIANS' MUTUAL BENEFIT ASSOCIATON OF TEXAS.

There are three deaths reported amongst the members since the last assessment (No. 8, benefit Dr. F. A. Tompkins certificate) to-wit: Drs. W. F. Buck, late of Pecos; J. T. Y. Jameson, of Rusk, and L. T. Maddox, of Birdston. Assessments have not yet been sent out, for the reason that during the latter part of last year, there occurred four deaths; and assessments were sent out so rapidly that the members forfeited in large numbers—seriously threatening the integrity of the association. As an appeal had just been issued, it was hoped there would be fresh accessions to the order, and it was thought best not to tax the members with these three other assessments in such rapid succession, for fear more would drop out, but to "give them a rest" in the hope that they would more promptly respond.

The dues for 1889 having been paid by, probably, all who intend to pay, and some months having elapsed since the last assessment was issued, there will be no longer delay, but members may expect to be called on now very shortly for their pittance to the death benefit fund for the widows of the above named deceased members.

Payment of No. 8 was so slow that the small amount of $111 has just only recently been realized.

LIST OF THOSE WHO PAID NO. 8.

Daniel, F. E. Burts, Wolff, Sholars, Cupples, Herff, Swearingen, Day, Ducheine, Dupree, Towsey, Coffey, Matthews, Sterrett, Paine, Brown, Styles, Myers, Powell, Taylor, McLaughlin, Hardy, Weller, McFarland, Inabnit, Paulus, Webb, Gray, Ball, Reid, Strain, Tidwell, Hill, Morris, Bennett, Ross Francis, Thorpe, Thorckmorton, Hons, Scales, Perkins, Keating, Osborne, Ford, Smith, Q. C., Tinsley, Litten, McClintock, Fox, Jameson, W. G., Jameson, J. T. Y.; Martin, Gregg, Wilson, Evans, Frazer, Field, Wadgymar, Fennell, Shuford, Burke, Bowers, Patton & Patton, Denman, Talley, Lancaster, Dupree, Watlington, May, Getzwiller, McGehee, Taylor, R. A. Stinson, Fry, Evans, A. D., Bell, McCrary, Southworth, Witherspoon, Jones, Bass, Davies, Hamilton, Styles, Taylor, R. A.; Cain, Barbee, Nicholson, Starnes, Burrows, Vauter, Robinson, Bruce, Grace, Smith, Bat, Philips, J. T.; Felder, Harmer, Johnson, L. B., Matthews, Burroughs, Seymour, Oliver, Sams, Wilkes, Crawford, Stone, $111.

This amount, $111 less the cost of collecting, $5, has been sent to Mrs. Tompkins, by check on Galveston, but her receipt has not been received.

Dr. F. E. Yoakum, of Greenville, writes the secretary that he is getting up a list of members for the P. M. B. A., and will get every Doctor in that county. We hope others will do as well.

